# Labial and Palatine Cavities—Cause, Mode of Preparation, and with Whot to Fill Them

**Published:** 1888-09

**Authors:** H. F. Harvey

**Affiliations:** Cleveland, O.


ARTICLE IV.

### LABIAL AND PALATINE CAVITIES—CAUSE, MODE OF PREPARATION, AND WITH WHAT TO FILL THEM.

BY H. F. HARVEY, D. D. S., CLEVELAND, O.

[Read before the Northern Ohio Dental Association, May 8, 1888 ]

The etiology of dental caries has been in the past, and is still the subject of much thought in dental literature, and of much discussion in the various societies throughout the country.

The subject is one that demands and is receiving the attention of the most scientific men in the profession, and the least I could hope for or the most that I can expect to accomplish, is to comply with the programme as prepared by the Executive Committee (without consulting the author) and bring this question before the society for discussion. In view of the fact that this subject has already received so much attention, would it not be well at this point to consider a few reasons why this question has so long remained unsettled ?

In the first place it must be borne in mind that there are comparatively few men who are competent for scientific investigation.

As a profession we are not systematic enough in our methods of observation. We do not study our cases with the critical care that we ought, or take sufficient notes concerning them.

Investigations in this direction must be conducted by men who have spent much time in preparing themselves for the work, and until we can secure a number of such men, properly qualified to experiment scientifically, the question will likely remain unsettled, and even then time must elapse for their conclusions to be verified.

It is gratifying, however, to know that we have some such men and that progress is being made. Work in this direction involves so much time and labor that it should receive substantial encouragement by the profession through the various societies. There are so many important factors that enter into the consideration of this subject that the causes of decay are commonly divided into two classes, viz., predisposing and exciting. The former includes hereditary, race, food, and climatic influences, modes of living, mental strain, lack of pure air and proper exercise, overtaxed nervous system, non-assimilation, and in fact all pathological conditions, the result of which is inferior structural development of the teeth, and the lack of power to resist the attack of what may be considered as the exciting causes. Under this latter class there seems to have been an effort on the part of investigators to find an active agent or a specific cause that would account for all decay in the human teeth.

Many theories have been advanced from time to time which vary as they are viewed from the scientific standpoint of the pathologist, chemist, electrician, or bacteriologist. At present the latter seems to be meeting with most favor, and according to this theory the micro-organisms take a very active part in the destruction of the human teeth. The experiments of Dr. Miller seem to have been conducted in a very scientific manner, (a fact which must carry weight with his conclusions,) and to quote briefly from him: "Decalcification is produced chiefly by acids resulting from the action of micro-organisms upon the carbo-hydrates in the human mouth, while the peptonization is produced either by the direct action of the protoplasm of the organisms upon the decalcified dentine or by a ferment which they produce." And further, he claims to have proven by different chemical analysis that the special acid concerned is lactic. A neutral or slightly alkaline condition of the mouth is normal. An acid condition is pathological. This pathological condition of the secretions of the mouth frequently accompanies some form of general disease and is often observed in parturition. Acid foods and medicines doubtless play an important role in decay of the teeth, not only locally but acting through the system. Local diseases of the gums and mucous membranes of the mouth often give rise to acidity of the secretions. Associated with these conditions we most frequently find the class of cavities designated by the title of this paper. These cavities usually appear at or near the margin of the gum, and occurring as they frequently do in teeth that otherwise seem perfect in structure, would indicate that the immediate cause or causes might have a different origin from that which produces decay in the crown of molars where there is defective enamel formation, or approximal surfaces where contact is a factor to be considered. Whether the exciting cause of these cavities is to be found in a specific that is common to all diseases in the teeth or whether they are the result of different influences, I leave for this society to discuss.

What we term erosion though most frequently occuring within the territorial limits of this paper can hardly be included in it, and for a very able article on this subject I would refer you to a paper published in the April number of the *Independent Practitioner*, 1886, by Dr. E. D. Swain, of Chicago. He leaves the subject for others to answer. I must be satisfied to close the first part of this paper in the same manner and proceed to a brief consideration of the second division of this subject. "The mode of preparation." This can be demonstrated clinically better than told, and as there is a clinic of this kind on the programme I will but briefly state a few principal points which of course must be modified as the necessities of a special case demand.

If the cavity extends much under the margin of the gum I first apply an 8 or 10 per cent. solution cocaine to lessen both pain and hæmorrhage in securing the rubber dam in position. To secure dryness and access to the cavity are the first important points and not always easy to accomplish. The clamp that I find most satisfactory for the majority of labial cavities is Dr. Hodson's, No. 71, White's catalogue. This I modify by grinding the posterior part to about one-eighth of an inch in width. After forcing it to the required position place a small wooden wedge between the anterior portion of the clamp and labial surface of tooth near the cutting edge. This will hold the clamp more firmly in position, and the loop being broad gives an opportunity to gain access to the cavity from all directions. The devise of Dr. Barnes, will be found useful in special cases. After removing more or less of the decay with an excavator, use a fissure bur with square end and size in proportion to cavity, carry this at right angle with the axis of the tooth, thus forming floor and margin of the cavity at the same time. Make slight undercut with hoe-shaped excavator or small wheel shaped bur and bevel the edges slightly with small finishing bur. There being no special strain upon these fillings a slight anchorage is sufficient. As regards the selection of a material for filling, circumstances must influence the operator in his choice for special cases. The extent of decay, quality of tooth structure, physical condition of patient, age, probable time that the tooth will be retained and location of cavity, are among the prominent points to be considered in deciding this question. For labial cavities gold is the standard, and gold or tin for the palatine. When plastic fillings are resorted to, gutta percha or Hill's stopping should be used in preference to the cements where the cavities extend far beneath the margin of the gum, as the agents that cause these cavities seem to dissolve the cements.

DISCUSSION.

*Dr. C. R. Butler:* There are some good suggestions in the paper in regard to the preparation of these cavities. First, the securing of good access and dryness; and second, in the manner of cutting the cavity for the retention of the filling material. Many cavities can be cut in the main with excavators alone and often quicker and better than with the engine. There is no necessity for deep undercuts, but cut to such an angle that the gold will be retained until we get the filling securely anchored. This procedure is best in these cavities for when once inserted we have no masticating force to dislodge the filling. Often the cutting of pits is the most painful portion of our operations and if we can avoid this we should.

*Dr. J. H. Siddall* advised the younger men in the profession to witness operations of the older members and gain all they could.

*Dr. H. Barnes* had noticed in the labial cavities especially, that where operations have failed, the decay in many cases has extended upward beneath the gum, and realizing this fact designed a clamp and matrix that would fit up under the gum and not lacerate it. After applying the matrix to the tooth if any portion of the rubber-dam extends into the cavity he dissolves it out with a little chloroform. The exposition of the cavity is perfect and an advantage gained in having a matrix that can be pushed from side to side and out of the way, on account of its pliability. This could be successfully used in many cases where clamps would not do. In regard to cements he had heard that some of the German makes were superior to our own, and if such is the case we should use them.

*Dr. Butler:* It is not the filling that fails but the tooth substance. How shall we treat these cases? He cited a case that came under his observation, a short time previous, where some of the teeth had been filled entirely with gold and others filled at the cervical border with tin and the rest of the cavity with gold thus leaving a dark line, perhaps the thickness of card-board, along the margin of the filling. These fillings, however, were preserving perfectly while most of the all gold fillings leaked. He further stated that very often this line can be covered by the gum and that we can get a good effect by this use of tin. Thus can we not lay aside the idea of some little disfigurement of color for the sake of good preservation?

*Dr. Siddall* spoke of a case where two kinds of metals had been used by the dentist and the patient complained of a peculiar sensation in the tooth thereafter. He inquired if it was due to the formation of an electric current?

*Dr. Bethel* inquired if the doctor did not find that in such cases the two metals were always separated and the gum or other tissue formed a conductor? As it is generally understood that where two metals are brought into immediate contact the electric current is not established.

*Dr. Dewey* said there must be some intervening substance to act as a conductor or no current will be established. In answer to Dr. Butler he thought that we can help check the decay in this situation by correcting the faults of the patients. That we have patients who take extra care of their teeth as far as brushing is concerned, but are at fault sometimes in eating sweets especially at night, and not perfectly cleansing the teeth thereafter. He had noticed, particularly in the mouths of servants and confectioners, that a white line of decay about the necks of teeth, was often very marked.

*Dr. Barnes* had noticed that the teeth on the side on which people slept most, were more decayed than upon the opposite side of the mouth.

*Dr. Fowler* said, three years ago I excavated two cavities, a cuspid and bicuspid, on the lower jaw. Filled the cuspid with gold when the patient said she could not afford gold in both so filled the bicuspid with tin. A few weeks ago the patient returned and I found the gold filling undermined while the tin was preserving perfectly.

*Dr. Butler* inquired how Dr. Fowler used tin foil.

*Dr. Fowler:* Fold it in squares and make pellets; never roll.

*Dr. Butler:* By using direct and then lateral force in working tin you get a stronger mass than by direct pressure alone. By ruffling the sheet of tin before using you loosen the fibres and it works much more satisfactorily.

*Dr. Bell* was averse to using tin exclusively. He prefers tin and gold used by the Brophy method.—*The Ohio Journal of Dental Science.*

